# Implications of Inflammatory States on Dysfunctional Immune Responses in Aging and Obesity

**DOI:** 10.3389/fragi.2021.732414

**Published:** 2021-09-22

**Authors:** Alyssa L. Thomas, Pablo C. Alarcon, Senad Divanovic, Claire A. Chougnet, David A. Hildeman, Maria E. Moreno-Fernandez

**Affiliations:** ^1^ Department of Pediatrics, University of Cincinnati College of Medicine, Cincinnati, OH, United States; ^2^ Division of Immunobiology Cincinnati Children’s Hospital Medical Center, Cincinnati, OH, United States; ^3^ Immunology Graduate Program and Medical Scientist Training Program, Cincinnati Children's Hospital Medical Center and The University of Cincinnati College of Medicine, Cincinnati, OH, United States; ^4^ Medical Scientist Training Program, Cincinnati Children’s Hospital Medical Center and The University of Cincinnati College of Medicine, Cincinnati, OH, United States; ^5^ Center for Inflammation and Tolerance, Cincinnati Children’s Hospital Medical Center, Cincinnati, OH, United States; ^6^ Center for Transplant Immunology, Cincinnati Children’s Hospital Medical Center, Cincinnati, OH, United States

**Keywords:** aging, obesity, inflammaging, anti-inflammation, immune system, immunosenecence, chronic inflammation

## Abstract

Aging and obesity are two conditions characterized by chronic, low-grade inflammation. While both conditions are also associated with dysfunctional immune responses, the shared and distinct underlying mechanisms are just starting to be uncovered. In fact, recent findings have suggested that the effects of obesity on the immune system can be thought of as a state of accelerated aging. Here we propose that chronic, low-grade inflammation seen in obesity and aging is complex, affects multiple cell types, and results in an altered basal immune state. In aging, part of this altered state is the emergence of regulatory immune populations that lead to further immune dysfunction in an attempt to reduce chronic inflammation. While in obesity, part of the altered state is the effect of expanding adipose tissue on immune cell function. Thus, in this review, we compare, and contrast altered immune states in aging and obesity and discuss their potential contribution to a shared clinical problem- decreased vaccine responsiveness.

## Introduction

First described in 2000, both chronic, low-grade inflammation, known as “inflammaging,” and age-related changes in the immune system, known as immunosenescence, are now recognized as hallmarks of the defective aging immune system (Franceschi et al., 2000a; Franceschi et al., 2000b). Inflammaging and immunosenescence create a balancing act that the aging immune system must contend with. These states are associated with increased rates of frailty, cardiovascular disease, Alzheimer’s disease, and susceptibility to infection (Giunta et al., 2008; North and Sinclair, 2012; Monti et al., 2017; Leonardi et al., 2018). Likely as a counter-response to low-grade chronic inflammation, we and others have recently demonstrated that aging also promotes the accumulation of anti-inflammatory cells and molecules, which in turn shape the landscape around age-related immune suppression (Sharma et al., 2006; Almanan et al., 2020).

Chronic inflammation is also a hallmark of obesity. Obesity-associated chronic inflammation is pathophysiologically linked to a variety of adverse sequelae, including metabolic syndrome, type II diabetes (T2D), dyslipidemia, non-alcoholic fatty liver disease (NAFLD), cardiovascular disease, Alzheimer’s disease, and diverse cancers (Calle et al., 2003; Weisberg et al., 2003; Strissel et al., 2007; Schenk et al., 2008). Like in aging, extensive characterization of the immune system in obesity has also revealed alteration of anti-inflammatory mechanisms (Pirola and Ferraz, 2017; Liu and Nikolajczyk, 2019), likely in response to obesity-driven low-grade chronic inflammation.

The immune system in obesity has been posited to display an “immunosenescence” phenotype, similar to that seen in aging (Shirakawa et al., 2016; Salvestrini et al., 2019), where cells become more broadly inflammatory even though their cell-specific functionality is altered. Nonetheless, parallels between the elevated basal rate of inflammation in aged and obesity-related inflammation remain insufficiently explored. It can be posited that the immune cells in aging and obesity become more inflammatory as a manner of trying to compensate for their loss of more specific and targeted functionality. Although immune systems of aged and obese individuals are similar in their inflammatory state, immunomodulatory responses of the immune system are different, suggesting aging and obesity use differing programs to quell the onslaught of inflammatory mediators. Though the pro- and anti-inflammatory responses differ somewhat in aging and obesity, they lead to similar outcomes inclusive of immune system dysfunction.

**FIGURE 1:**
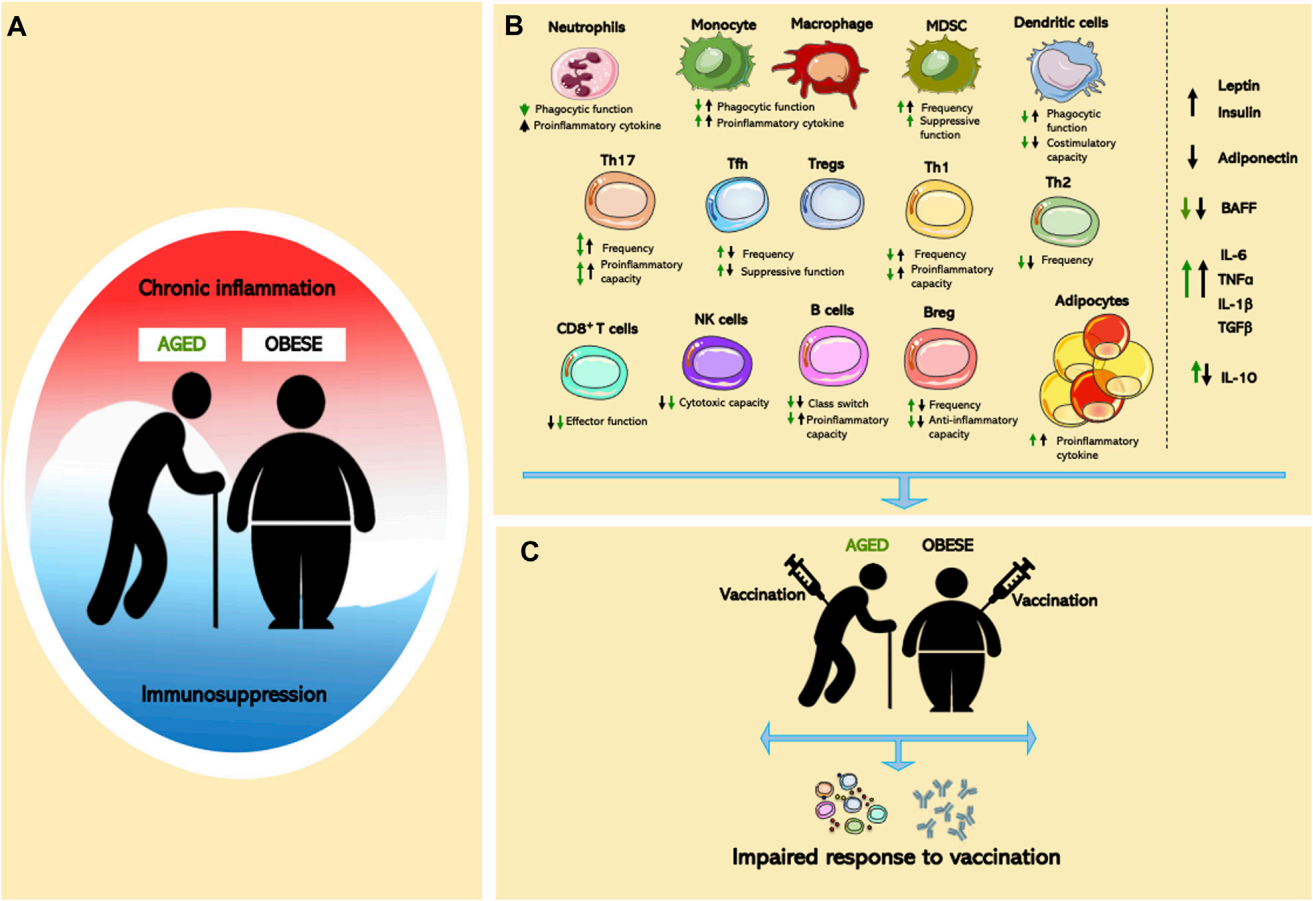
Effects of aging and obesity on immune system. **(A)** Chronic inflammation and immunosuppression are a dynamic process in aging and obesity. **(B)** Cellular dysregulated adipocytes, innate/adaptive immune cells, and pro- and anti-inflammatory mediators in aged or obese individuals lead to global immune cell dysfunction. **(C)** Immunological dysfunction contributes to impaired vaccine responses in age and obesity.

Notably, the mechanistic similarities and differences between the two chronic inflammatory states remain poorly understood, especially when the two conditions co-exist. In this review, we evaluate and compare inflammatory states of cells and mediators that compromise the aging and obese immune system. For simplicity, we describe their role in obesity and aging as either pro- or anti-inflammatory (Figure 1), although in reality inflammation exists on a sliding scale where each cell or mediator can have either pro- or anti-inflammatory roles given the right context. Finally, we compare the impact of altered immune states in both aging and obesity, utilizing decreased vaccine responsiveness as an exemplar. Furthermore, the underlying immune mechanisms linked to immune dysfunction in aging and obesity are likely to impact many other diseases associated with aging and/or obesity.

### Aging-Associated Chronic Inflammation

Inflammaging is the chronic low-grade inflammation associated with aging (Franceschi et al., 2000b). Multiple age-associated diseases including cardiovascular diseases, neurodegenerative diseases, and various cancers are largely driven by this chronic inflammation (Giunta et al., 2008; North and Sinclair, 2012; Monti et al., 2017; Leonardi et al., 2018). Key inflammatory markers (e.g., Interleukin-6 [IL-6]) are elevated in aged humans and mice and contribute to inflammaging (Wei et al., 1992; Giuliani et al., 2001; Raynor et al., 2015). There are multiple theories surrounding the underlying mechanism and cellular source of increase inflammaging-associated inflammatory mediators. Senescent cells, obesity, increased gut permeability, changes to microbiota, inflammasome activation, oxidative stress caused, and chronic infections are likely major contributors. For instance, upon continual cellular stress (e.g., persistent DNA damage signaling, accumulation of reactive oxygen species [ROS], failure to remove defective cellular components) cells can exhibit senescence associated secretory phenotype (SASP), characterized by cellular growth arrest and a pro-inflammatory secretory phenotype (van Deursen, 2014). Senescent cells secrete many inflammatory mediators (e.g., IL-6, Interleukin-1 [IL-1] and colony stimulating factor [CSF]) (Garfinkel et al., 1994; Coppé et al., 2008), thus their accumulation might be directly responsible for systemic increases in inflammatory proteins with age. In addition, stressed senescent cells produce damage-associated molecular patterns (DAMPs) further activating immune cells pro-inflammatory programming (Huang et al., 2015). Moreover, persistent infections acquired throughout an individual’s lifespan exert a constant pressure on the immune system (Brunner et al., 2011; Minciullo et al., 2016). However, whether the presence of persistent infections contributes to age-related immune senescence remains under debate (Nikolich-Žugich, 2008).

Overall, immune cell functionality declines with aging while exhibiting a bias towards pro-inflammatory phenotypes, further contributing to inflammaging. Aging is associated with a shift in myeloid cells output by the bone marrow; however, these myeloid cells are largely dysfunctional (Pang et al., 2011). Published reports have shown that aged human monocytes shift to a more pro-inflammatory phenotype that correlates with increased production of IL-6, tumor necrosis factor (TNF), and IL-1β compared to their younger counterparts (Sadeghi et al., 1999). Also, neutrophil (Wenisch et al., 2000) and macrophage (Linehan et al., 2014; Wong et al., 2017) phagocytosis decreases with age, preventing bacterial clearance and timely and efficient removal of cellular debris in the case of injury or even normal tissue homeostasis. This results in increased prevalence of DAMPs that further amplifies pro-inflammatory pathways. Similarly, dendritic cell (DC) populations are altered in aging. Aged DCs are less phagocytic and less efficient in the cross-presentation of cell-associated antigens and subsequently in the cross-priming of CD8^+^ T cells and subsequent effector responses compared to their younger counterparts (Nikolich-Žugich et al., 2012; Chougnet et al., 2015). The aging T cell compartment produces more inflammatory cytokines compared to their younger counterparts (Fagiolo et al., 1993), and the involuted thymus produces more autoreactive T cells, which are also known to be more inflammatory (Coder et al., 2015). Furthermore, circulating CD4^+^ T cells from aged individuals display an increased Th17 associated cytokine profile, driven by increased mitochondrial dysfunction and ROS production (Bharath et al., 2020). Together these findings suggest that dysfunction in both the innate and adaptive immune compartments contribute to the inflammaging and immune dysfunction in the elderly.

### Obesity-Induced Chronic Inflammation

Similar to chronic inflammation in aging, the obese chronic inflammatory state is central to the development of obesity-associated sequelae (e.g., T2D, and NAFLD) (Whitlock et al., 2009; Gregor and Hotamisligil, 2011). While obesity impacts circulating immune cells in both pro- and anti-inflammatory manners, nearly all the cellular populations present within adipose tissue develop an inflammatory phenotype (Elgazar-Carmon et al., 2008; Nishimura et al., 2009; Bertola et al., 2012; Talukdar et al., 2012; Shaikh et al., 2015; Wensveen et al., 2015; Lauterbach and Wunderlich, 2017; Russo and Lumeng, 2018; Zhao et al., 2018). Hence, it is not surprising that adipose tissue expansion is considered the key initiator of inflammation in obesity. The adipose tissue-associated inflammatory microenvironment shapes the chronic, low-grade inflammation in obesity with both adipose tissue-resident immune cells and inflammatory-skewed adipocytes contributing to the overall effect (Fontana et al., 2007; Reilly and Saltiel, 2017; Chan et al., 2020; Alarcon et al., 2021). Changes in the adipose tissue microenvironment result in increased volume of inflammatory cells and subsequent secretion of inflammatory mediators (e.g., Leptin, IL-6, IL-1β) (Skurk et al., 2007; Strissel et al., 2007; Grant and Dixit, 2015; Wueest and Konrad, 2018). Increased levels of these inflammatory mediators may also contribute to decreased adaptive immune function as plasma from obese individuals is sufficient to induce senescence in cytotoxic T cells (Parisi et al., 2017). Of note, as adipose tissue distribution varies by race (Stults-Kolehmainen et al., 2013), such changes could contribute to observed differences in immune responses between obese individuals of diverse races and ethnicities.

The impact of obesity on the innate immune compartment has been the most studied thus far. Published reports have shown increased total numbers of circulating monocytes, macrophages, and neutrophils in obesity (Roberts et al., 2018; Friedrich et al., 2019). Further, these innate immune populations are skewed towards a pro-inflammatory state, with macrophages being skewed towards an inflammatory phenotype and neutrophils having elevated cytokine, reactive oxygen species, and extracellular trap formation, contributing to elevated circulating levels of proinflammatory cytokines (e.g., IL-6, TNFα) (Lumeng, 2013; D'Abbondanza et al., 2019; de Heredia et al., 2012). However, obesity doesn’t impact all immune cells equally. Although increased numbers of DCs are observed in obesity, the chronic inflammation leads to impaired responsiveness of DCs to toll-like receptor (TLR) agonists (Pizzolla et al., 2016). The obesity-driven inflammation also negatively impacts effector function of other innate cells (e.g., NK cells), decreasing their total numbers and their cytotoxic potential (O'Shea and Hogan, 2019; Viel et al., 2017). Intriguingly, the impact of obesity on splenic NK cells closely resembles an aging-induced immunosenescent state (Gheorghe et al., 2017), further supporting the concept of obesity induced “accelerated aging” phenotype (Salvestrini et al., 2019).

Recent studies have also begun to explore obesity’s impact on adaptive immune cell function. Obesity-driven inflammation is also associated with decreased proliferation capability of naïve T cells (James et al., 2012). CD4^+^ helper T cells are skewed towards inflammatory subtypes, with increased Th17 and Th1 (McLaughlin et al., 2017; Moreno-Fernandez et al., 2021a) and decreased Th2 and Treg polarization (Zhao et al., 2018). CD8^+^ T cells are impacted similarly to NK cells, with decreased numbers and limited cytotoxic potential (Kado et al., 2019). In animal models of obesity, increases in adiposity and leukocyte infiltration that occur in the bone marrow negatively impact B cell bone marrow precursor populations (Adler et al., 2014). This results in B cells being skewed towards an inflammatory phenotype (Frasca et al., 2017a). This inflammatory skewing may lead to an increased population of exhausted memory B cells (Frasca et al., 2016; Frasca et al., 2017b), invoking the possibility that obesity-induced inflammatory states further exacerbate deficiencies in antibody responses and subsequently vaccine unresponsiveness. Importantly, B cells accumulate in the adipose tissue as a result of obesogenic diet (Duffaut et al., 2009; Winer et al., 2011). However, in human subcutaneous and visceral adipose tissue only a small fraction of B cells is observed (McDonnell et al., 2012; García-Rubio et al., 2018). Low frequency of B cells can likely be a consequence of low density of fat-associated lymphoid clusters in omental and subcutaneous human adipose tissues, which are structures associated with B cell accumulation Notably, B cell depletion improved insulin and glucose responses in obesity/T2D, which correlated with decreased inflammation (DeFuria et al., 2013). However, more work is required to mechanistically link chronic adipose tissue inflammation driven by the adaptive immune system to local and systemic immune cell dysfunction/senescence in human disease.

### Aging-Associated Immunological Regulation

While aging and obesity are associated with markers of chronic inflammation, both states also lead to altered immunological regulation. Recent work, including findings from our group, have shown that alongside chronic inflammation activation of immune regulatory mechanisms and secretion of anti-inflammatory mediators are exacerbated in aging. Aged mice show an increase in myeloid-derived suppressor cells (MDSCs) in the spleen and peripheral lymph nodes (Heithoff et al., 2008; Enioutina et al., 2011). These aged MDSCs exhibit greater capacity to suppress T cell proliferation and cytotoxic function compared to their younger counterparts (Grizzle et al., 2007; Enioutina et al., 2011). Additionally, macrophages with anti-inflammatory characteristics, including increased secretion of Interleukin-10 (IL-10) and Transforming Growth Factor Beta (TGFβ) are more frequent in aged bone marrow, lymph nodes, and skeletal muscle (Jackaman et al., 2013; Wang et al., 2015).

Regarding the B cell and T cell compartment, regulatory B cells (Bregs) have been shown to increase in aging and conserve their suppressive function by producing similar IL-10 levels compared to young Breg cells (Mori et al., 2016; Freitas et al., 2019). Further, we and others have shown that, in mice and humans, regulatory T cells (Tregs, CD4^+^FOXP3^+^) accumulate with age (Nishioka et al., 2006; Sharma et al., 2006; Lages et al., 2008; Raynor et al., 2012). IL-6 drives the accumulation of Tregs suggesting this is a compensatory pathway attempting to dampen the chronic low-grade inflammation associated with aging (Raynor et al., 2015). Aged Tregs can 1) suppress the activation of DCs via decreased CD86 expression, 2) enhance suppression of effector T cell proliferation, and 3) secrete more IL-10 compared to younger counterparts (Garg et al., 2014). However, aged Tregs fail to suppress Th17 cells’ production of IL-17 during autoimmune inflammation (Sun et al., 2012). Together these data suggest that aging Treg have an altered functional profile.

More recently, we show that IL-10 actively suppresses vaccine responses in aged mice as neutralization of IL-10 restored antigen-specific antibody levels to nearly those observed in young mice (Almanan et al., 2020). In this study, the greatest producer of IL-10 in aged mice was a novel population of T follicular helper cells, which we called Tfh10 cells. These Tfh10 cells appear to regulate the systemic IL-6:IL-10 balance which is crucial to healthy aging (Monti et al., 2017; Almanan et al., 2020). Our data also showed that IL-10R blockade resulted in an increase in antigen-specific, germinal center B cells (Almanan et al., 2020), suggesting this accumulation of Tfh10 cells in aging is drastically dampening B cell responses. These B cell responses are vital for producing strong antibodies in both a vaccine and infection setting, both of which significantly decrease with age (Frasca and Blomberg, 2011; Simell et al., 2011; Frasca and Blomberg, 2020). Combined, our data suggest that there is active immune suppression in aging, reversable by neutralization of a single cytokine, IL-10, which is sufficient to restore antibody responses in aged mice. Another IL-10 cellular source is T follicular regulatory (Tfr) cells, a novel CD4^+^ T cells population that are FoxP3^+^ and Bcl6^+^ and express high levels of PD-1 and CXCR5, have been shown to be critical for regulating germinal center B cell reactions such as plasmablast formation, affinity maturation, and class switching (Frasca et al., 2004; Frasca et al., 2011). Tfr cells frequency was reported to be increased in aged mice and humans (Sage et al., 2015; Lefebvre et al., 2016a). Although, at the cellular level Tfr cells were found to display impaired suppressive function due to their age-related decrease in IL-10 production (Lefebvre et al., 2016a; Ito et al., 2019), their suppressive function in aging is associated with expansion. Indeed, we recently reported increased serum IL-10 levels in aged mice (Almanan et al., 2020).

Although inflammaging is well characterized, there is evidence that both pro- and anti-inflammatory immune programs are present in aging, thus a more detailed investigation of how the anti-inflammatory arm of the immune system is regulated in aging and how it interacts with age-associated chronic inflammation is needed. Logically, the overabundance of pro-inflammatory mediators that overwhelm an aged individual is bound to elicit a “brake” response, by upregulating anti-inflammatory cells and mediators. Thus, an improved understanding of the interplay of pro- and anti-inflammatory immune programs in aging may provide important insights into overall immune function and potential revitalization in aging.

### Obesity Induced Alteration in Anti-inflammatory Mediators

Obesity is also associated with alterations in multiple types of immunoregulatory cells and mediators, including Tregs, Bregs, MDSC, anti-inflammatory macrophages and IL-10. While such immune changes plausibly contribute to the persistent low-grade immune activation associated with the obesogenic state, how such changes are modified in elderly obese and the contribution of their effects on metabolic disease remains unclear.

Treg frequency declines in the peripheral blood in obese humans (Cortez-Espinosa et al., 2015; Yuan et al., 2018) and in the adipose tissue (Wu et al., 2019; Smith et al., 2020). Furthermore, metabolic decline in individuals newly diagnosed with obesity-driven T2D is associated with reduced circulating Treg frequencies (Yuan et al., 2018). In animal models of obesity driven metabolic disease, in homeostatic condition (lean state), Treg cell numbers are expanded in adipose tissue compared to the obesogenic state, where Treg frequency decreased (Feuerer et al., 2009; Bapat et al., 2015). Treg cells play a protective role in insulin sensitivity and energy homeostasis in obesity (Ilan et al., 2010). Increased adipose inflammation was observed in Treg-depleted mice and altered glucose metabolism was ameliorated in obese mice after adoptive transfer of Treg cells (Feuerer et al., 2009; Eller et al., 2011). As Treg frequency differs in a gender dependent manner (Ishikawa et al., 2020; Vasanthakumar et al., 2020), the impact of obesity on Treg homeostasis may differ between genders. Of note, female mice display lower visceral adipose tissue Treg frequency than male mice (Vasanthakumar et al., 2020). This was associated with estrogen levels, as visceral adipose tissue Tregs frequencies increases in estrogen receptor alpha-depleted or testosterone-treated female mice (Vasanthakumar et al., 2020). Treg accrual is also reduced in adipose tissue in obese male mice compared to lean animals (Feuerer et al., 2009; Ishikawa et al., 2020). In contrast, obesogenic diet feeding promoted adipose tissue Treg expansion in female mice (Ishikawa et al., 2020), which was associated with limited induction of metabolic diseases (Moreno-Fernandez et al., 2021b). Thus, change in female sex hormones during aging may be implicated in altered adiposity and Treg frequency in the context of increase adiposity during aging. Further, the above mentioned hormonal effect on the immune system may explain lower propensity of females to age-related metabolic alterations in the elderly.

In addition to altered Treg homeostasis, Treg function is impacted in obesity. Numbers of Tregs expressing CD39, an immunomodulatory ecto-5′- nucleotidases, decline in obese individuals who have T2D (Cortez-Espinosa et al., 2015). Importantly, high levels of expression of PD1 and T cell immunoreceptor with Ig and ITIM domains (TIGIT) is observed on Tregs as well as other CD4^+^ T cells in the adipose tissue of obese humans and mice (Smith et al., 2020; Porsche et al., 2021). However, the role of PD1 pathway is not clear, since PD1 blockade did not affect T cell function and metabolic alteration in obese mice (Porsche et al., 2021). These data suggest that although T cells PD1 is increased additional mechanisms may contribute to T cell exhaustion in obesity.

Increased insulin levels in obesity, a consequence of insulin resistance, may play an important role in shaping Tregs function. Treg sensing of insulin is important as deletion of the insulin receptor in Tregs improved glucose tolerance and insulin sensitivity and increased numbers of IL-10 producing Tregs in adipose tissue of obese animals (Wu et al., 2020). Additionally, insulin administration decreased IL-10-expressing Tregs and diminished Treg capacity to suppress macrophage function (Han et al., 2014). Similarly, levels of leptin, an adipokine, remain high during obesity as a consequence of leptin resistance. Leptin can skew the T cell balance towards an inflammatory state favoring Th17 cell differentiation (Reis et al., 2015) at the expense of Tregs. Hence, circulating leptin levels inversely correlate with circulating Tregs frequency (Wagner et al., 2013). In contrast, levels of adiponectin, another adipokine, are decreased in the obese state (Musovic and Olofsson, 2019). Adiponectin has anti-inflammatory properties that limit production of reactive oxygen species and Th1 cell polarization (Robinson et al., 2011). Combined, these data suggest that increased insulin and leptin in conjunction with decreased adiponectin in the context of obesity likely shifts the balance towards a more pathogenic/pro-inflammatory environment. These responses may influence Treg differentiation and further promote the accrual of inflammatory T cells.

Bregs are also impacted by obesity. Bregs, a subpopulation of B cells characterized by IL-10 production, play a critical role in the differentiation and maintenance of Tregs and in the suppression of T cell responses (Mizoguchi et al., 2000). Obese individuals have a decreased circulating Breg frequency (García-Hernández et al., 2018). In animal models, Breg numbers are decreased in the adipose tissue of obese animals compared to lean controls. Bregs restrict adipose tissue inflammation and insulin resistance in obese mice in an IL-10 dependent manner (Nishimura et al., 2013). Adoptive transfer of adipose tissue Bregs ameliorated those effects and maintained metabolic homeostasis in the lean adipose tissue (Nishimura et al., 2013). Additionally, CD40 or BCR stimulation of purified B cells from obese/T2D subjects led to decreased IL-10 production (Zhai et al., 2016). These data suggest that the impaired ability to secrete IL-10 and TGFβ by B cells in obese and T2D individuals could be linked to decreased Breg numbers and function in obesity and increased overall inflammation. Of note, reduced serum IL-10 levels are observed in patients with obesity-driven T2D (Yuan et al., 2018). IL-10 is a protective factor against diet-induced insulin resistance in the liver and in skeletal muscle as it attenuates macrophage cytokine secretion (Hong et al., 2009). Thus it would be important to determine whether increased IL-10 levels in aging, may play a beneficial role in the context of obesity-driven metabolic disease by ameliorating disease severity (Moreno-Fernandez et al., 2021b). In addition, B cell function is linked with leptin and BAFF levels (Frasca and Blomberg, 2020). BAFF levels are decreased in aging and in obese mice (Jin et al., 2008; Kim et al., 2009). Of note, increased BAFF levels has been implicated in the modulation of weight gain in mice and humans (Chan et al., 2021). Given that aged and obese individuals exhibit reduced antibody responses, the potentially unifying role of BAFF in aging and obesity should be further explored. Likewise, Breg development in obesity could be affected by the impact of obesity in Tfh cells (Garner-Spitzer et al., 2020). A recent study revealed that bariatric surgery (and subsequent weight loss) resulted in an increase of less inflammatory Tfh cells that had better capability to promote the development of Bregs (Zhan et al., 2017), suggesting that Tfh function during obesity is shifted, which may act as a rheostat that regulates overall inflammation.

Contrary to their other immunoregulatory counterparts, MDSC are poorly studied in obesity. Increased frequency of monocytic CD11b^+^CD33^+^CD14^+^HLADR^low/-^ MDSC was observed in the peripheral blood of obese individuals (Bao et al., 2015). In mouse models of obesity, a MDSC population expressing Gr-1 and CD11b is highly enriched in the liver and adipose tissue. This particular MDSC population suppressed CD8^+^ T cell and inflammatory macrophage function in obesity (Xia et al., 2011), suggesting that MDSC may act as another important counter regulatory mechanism of exacerbated inflammatory immune responses in obesity. Overall, though aging and obesity’s effect on immunomodulatory regulation differ, they both are associated with immune cell dysfunctionality, leading to abnormal immune responses.

### Inflammation-Induced Immunomodulation: Linking Aging and Obesity

Intriguing unifying parallels between aging- and obesity-dependent impact in the ability of the immune system to properly function and leading to dysfunctional responses warrant further investigation. Examining these trends is critical for understanding the differences and similarities between the associated-pathologies shared by both states. Of significant interest is the intersection of these two states and how immune alterations differ in those who are both elderly and obese in comparison to those that are only elderly or obese. However, many of the studies perform so far have largely focused on either individual state rather than on overlapping conditions. Therefore, which immunomodulatory program obese elderly individuals adopt, and how this helps them compensate for the compounded chronic inflammation are key questions that remain unanswered. Of note, use of thermoneutral housing allows for studying obesity and aging in unison in both male and female mice (Giles et al., 2017; Moreno-Fernandez et al., 2021b). Thus, this new model may allow for future interrogations of obesity and aging in both sexes and may lead to discovery of new underlying mechanisms linking obesity and aging.

One highly clinically relevant similarity between obesity and aging is their impact on vaccine-driven immune responses. Vaccines have saved hundreds of millions of lives by reducing disease mortality and morbidity. However, both elderly and obese individuals have decreased vaccine responsiveness. For example, yearly influenza vaccines provide 65–80% protection for young individuals while only about 30–50% protection to their older counterparts (Nichol et al., 2007). This lack of protection in aged individuals is largely attributed to age-related dysfunction in B and T cells that leads to a decline in antibody responses to the influenza vaccine (Goronzy et al., 2001; Frasca et al., 2010). Aged B cells accumulate intrinsic defects leading to decreased influenza-specific antibody titers, decreased induction of AID (activation-induced cytidine deaminase), a known inducer of Ig class-switch recombination and somatic hypermutation, and decreased memory induction (Frasca et al., 2010). Additionally, intrinsic defects in aged T cells have been implicated in the loss of influenza vaccine protection. Shifts in the aging CD4^+^ T cell compartment towards Tfh cells, instead of Th1 cells, have been proposed as a mechanism of decreased vaccine efficacy, as Th1 cells are required for effective influenza clearance in the lung (Lefebvre et al., 2016b). Additionally, as mentioned earlier excessive IL-10 production in aging suppresses vaccine responses in mice (Almanan et al., 2020). Other vaccines show similar decreased efficacy with aging. Hepatitis A and hepatitis B vaccines induce poor antibody responses in the elderly leading to a decrease in vaccine efficacy from 92% in young individuals to 63% for hepatitis A and 67–33% for hepatitis B respectively (Wolters et al., 2003). Additionally, the mRNA-1273 COVID-19 vaccine efficacy drops from 95.6% in individuals under 65 years old to 86.4% in those 65 years and older (Baden et al., 2021). Together, these studies suggest that the altered immune state in aged individuals severely limits their ability to produce adequate and appropriate immune responses, thus leaving these highly vulnerable populations without the protection that vaccines normally provide.

Similar to aged individuals, the impact of obesity on vaccine efficacy is well-reported (Painter et al., 2015). Obese individuals have decreased antibody levels and an overall faster decline in protective levels of antibodies than their lean counterparts for many vaccines including rabies (Banga et al., 2014), Hepatitis A (Reuman et al., 1997), Hepatitis B (Weber et al., 1985) or tetanus (Eliakim et al., 2006) vaccines. With regards to these normally highly efficient vaccines, obesity-associated chronic inflammation might detrimentally influence the longevity of adaptive immune cells that are known to be impacted by obesity. In addition, obese adults have double the risk of developing influenza-associated pneumonia despite having similar antibody titers to their lean counterparts (Green and Beck, 2017). In this case, worsened disease outcomes (e.g., impaired limitation of viral propagation, lung tissue pathology) have been attributed to the impaired cytotoxicity of NK cells in obese individuals (O'Shea and Hogan, 2019), and/or increased inflammatory propensity of lung macrophages and neutrophils (Narasaraju et al., 2011) in obese individuals. Contribution of obesity-altered adipocytes has also been evoked (Chan et al., 2019; Chan et al., 2020; Chan et al., 2021; Alarcon et al., 2021). This trend might be due to obesity-specific mechanisms, as T2D individuals have similar responsiveness to the influenza vaccine as non T2D individuals (Sheridan et al., 2015; Dos Santos et al., 2018). Nevertheless, it is important to consider these clinically relevant epidemiological trends, as they might have impact on current and future vaccination efforts, such as for SARS-CoV-2, where obese individuals are an “at risk” group (Popkin et al., 2020).

Although there is some evidence to indicate that aging with comorbidities such as obesity leads to worse outcomes in vaccine efficacy, the interplay between both obesity and aging have been largely understudied. Many reports of vaccine efficacy fail to report data of individuals that are both elderly and obese making it difficult to understand the respective and combined impact of these two states. However, recent preliminary studies have begun to include these analyses. For instance, it was suggested that the Johnson and Johnson COVID-19 vaccine efficacy 28 post vaccination could drop to 42.3% in elderly patients with comorbidities including obesity, compared to 72.4% efficacy in healthy elderly patients and 68.0% in healthy, young adults (Administration FaD, 2021). Hence the combinatory effects of both aging and obesity may exacerbate the worsened immune dysfunctional environment than either obesity or aging alone. Thus, further exploration and data analysis in this area are needed as the obese, elderly, and obese-elderly populations continue to increase.

Importantly, these clinical trends might be attributed to a unifying impact of aging- and obesity-inflammation-dependent skewing of the innate and adaptive immune response. However, we also acknowledge that while chronic inflammation appears to drive any similar immune phenotypes, differences do exist, likely driven by mechanism specific to each state. More research using multiomics approaches such as IMM-AGE study (Alpert et al., 2019) will be of great importance to decipher precise unique and share pathways activated in each individual or combine state over time. Further, uncovering of similarities and differences between these two states will also help to uncover whether anti-inflammatory treatment targeted towards amelioration of metabolic dysfunction could be exploitable for aging and/or aging/obesity associated inflammation and improving vaccine responses in these populations. For instance, metformin appears promising for treatment of chronic inflammatory diseases associated with both aging and obesity in pre-clinical models (Bharath et al., 2020). Notably, pioglitazone alone or in combination with metformin has also been shown to have anti-inflammatory properties (Zhang et al., 2008; Shen et al., 2018) and decrease inflammatory mediators in patients with T2D (Forst et al., 2008; Schöndorf et al., 2011). Undeniably, it is attracting to think about repurposing such drugs to improve vaccine efficacy in the elderly and/or obese. For example, preliminary studies of pioglitazone in experimental influenza infection improved survival by favoring protective and limiting exacerbated immune responsiveness in mice (Aldridge et al., 2009; Moseley et al., 2010). A better understanding of these mechanisms is thus imperative to provide better vaccine regimens for obese, elderly, or obese-elderly individuals.

## Concluding Remarks

Aging- and obesity-associated chronic inflammation and potential immunosuppression have a profound impact on the functionality of the immune system (Figure 1A). Both conditions exist as a mixed inflammatory state, where pro- and anti-inflammatory cells and mediators co-exist and contribute to the development of aging- and obesity-associated disease (Figure 1B). While similarities between the aged and obese immune systems have been noted, the literature concerning the impact of combined aged and obese state is limited, something that will be critically important to combat major health issues including vaccine efficacy (Figure 1C). Thus, further examination of the parallels between the aged and obese immune system are needed to identify critical inflammatory mechanistic links. Such studies might provide novel therapeutic approaches to ameliorate the clinical burden of disease in an increasingly obese world that continues to age.
